# Hybrid stenting through direct ventricular access for severe stenosis of the pulmonary venous baffle after atrial switch operation for transposition of the great arteries: a case report

**DOI:** 10.1093/ehjcr/ytae616

**Published:** 2024-11-20

**Authors:** Margaretha Van Kerrebroeck, Werner Budts, Agnès Pasquet, Pieter De Meester

**Affiliations:** Division of Congenital and Structural Cardiology, UZ Leuven, Herestraat 49, 3000 Leuven, Belgium; Department of Cardiovascular Sciences, KU Leuven, Herestraat 49 - box 911, 3000 Leuven, Belgium; Division of Congenital and Structural Cardiology, UZ Leuven, Herestraat 49, 3000 Leuven, Belgium; Department of Cardiovascular Sciences, KU Leuven, Herestraat 49 - box 911, 3000 Leuven, Belgium; Division of Cardiology, Cliniques Universitaires Saint-Luc, Avenue Hippocrate 10, 1200 Brussels, Belgium; Department of Cardiovascular Diseases, Pôle de Recherche Cardiovasculaire (CARD) Institut de Recherche Expérimentale et Clinique (IREC) Université Catholique de Louvain, Avenue Hippocrate 10, 1200 Brussels, Belgium; Division of Congenital and Structural Cardiology, UZ Leuven, Herestraat 49, 3000 Leuven, Belgium; Department of Cardiovascular Sciences, KU Leuven, Herestraat 49 - box 911, 3000 Leuven, Belgium

**Keywords:** Transcatheter intervention, Pulmonary venous baffle, Transposition of the great arteries, Mustard repair, Case report

## Abstract

**Background:**

Atrial switch repair was the first surgical intervention to result in long-term survival in patients with ventriculo-arterial discordance or transposition of the great arteries. However, the natural history after atrial switch is not uneventful with frequent atrial arrhythmia, development of baffle stenosis, and eventually heart failure. For this, new interventions might be necessary but are often associated with increased risk.

**Case summary:**

We present the case of a 49-year-old woman born with ventriculo-arterial discordance or dextro-transposition of the great arteries who underwent atrial switch repair according to Mustard at the age of 1 year. She presented with shortness of breath and reduced exercise capacity. The echocardiography revealed prominent turbulent flow at the level of the pulmonary venous baffle (PVB). This was confirmed on cardiac computed tomography. After multidisciplinary discussion, a hybrid approach was considered as the preferred strategy. In this, the cardiac surgeon provided apical access by left lateral thoracotomy. The PVB was accessed retrograde through right ventricular apical access, and stenting with a covered stent with subsequent balloon dilatation up to 13 mm was performed. This reduced the peak gradient on echocardiography from 18 to 11 mmHg. Clinical follow-up was uneventful with improved functional capacity 6 months after discharge.

**Discussion:**

This case provides an alternative access to the PVB by left lateral mini-thoracotomy and apical ventricular access. Furthermore, we highlight the challenges in decision-making and the importance of the multidisciplinary collaboration between adult congenital cardiologist, the echocardiographer, and cardiac surgeon as well as the flexibility in interventional techniques to individualize the management of such cases.

**Learning points**

- Atrial switch repair in ventriculo-arterial discordance patients leads to long-term complications including atrial arrhythmias, baffle leak or stenosis, and heart failure.
- Pulmonary venous baffle stenosis occurs in 3.8% of Mustard repair cases, often necessitating intervention due to symptomatic progression.
- A hybrid transcatheter approach is preferred over surgical re-intervention due to the patient’s complex medical history, high surgical risk, as well as the successful outcome and the feasibility of percutaneous stenting in reducing procedural risks.
- Accessing and effectively stenting the pulmonary venous baffle poses technical challenges, emphasizing the need for tailored interventional strategies.
- The use of a left lateral mini-thoracotomy and apical access facilitated successful stent placement and dilation, demonstrating an innovative approach.

## Introduction

Atrial switch repair (ASR) was the first surgical intervention to result in long-term survival in patients with ventriculo-arterial discordance or transposition of the great arteries. However, the natural history after ASR is not uneventful with frequent atrial arrhythmia, development of baffle stenosis, and eventually heart failure.^1,2^ Stenosis of the pulmonary venous baffle (PVB) is less common compared to systemic venous baffle stenosis, occurring with an incidence of about 3.8% in patients after Mustard repair.^2,3^ Two-thirds of these patients become symptomatic requiring re-intervention.^3^ Furthermore, the baffle is not easily approached through the systemic venous return. Hence, surgical redo ASR is often necessary, but peri-operative mortality has been reported to be as high as 36%.^3^ However, when access to the pulmonary venous atrium can be provided by the surgeon in a hybrid setting, balloon dilatation and stenting can be performed. Hybrid stenting has been described by means of subxiphoidal incision or ministernotomy, providing direct access to the pulmonary venous atrium. However, access through left lateral mini-thoracotomy and systemic ventricular access has not been published yet.^4,5^

## Summary figure

Stenosis of the PVB in a patient after ASR for transposition of the great arteries. Results after hybrid stenting via access through the systemic ventricular apex. PVB, pulmonary venous baffle; PVA, pulmonary venous atrium; sRV, systemic right ventricle.

**Figure:**
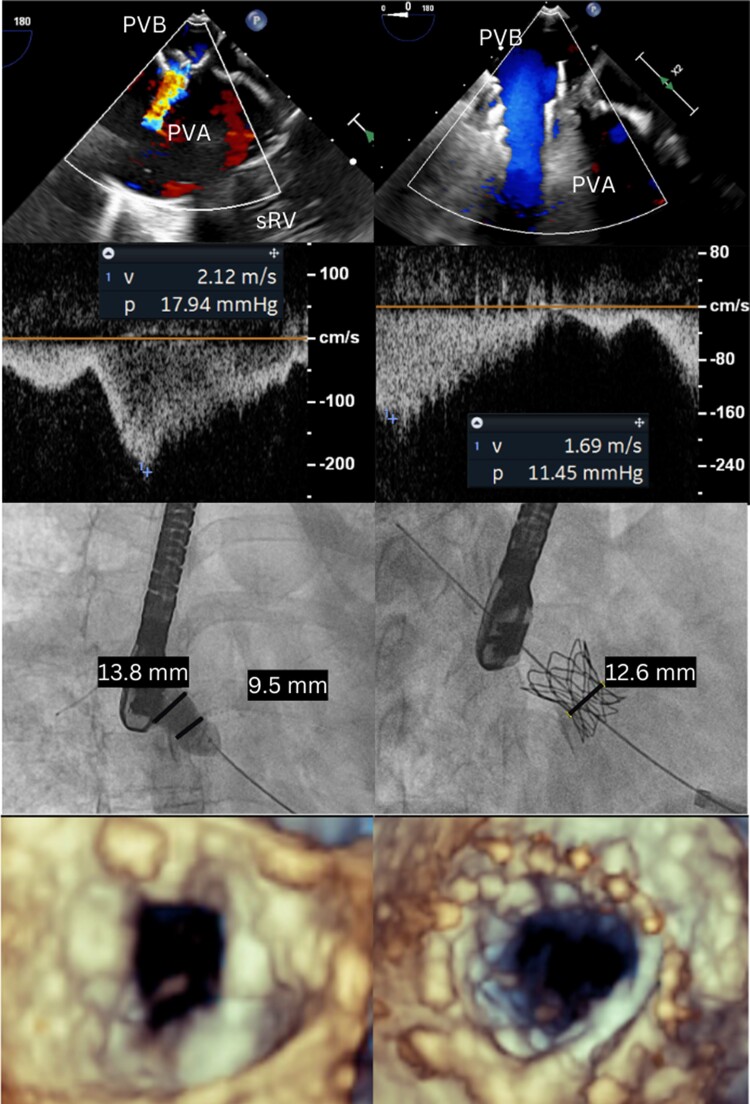


## Case presentation

A 49-year-old woman with complex congenital heart disease was referred to our hospital. The patient was born with ventriculo-arterial discordance or dextro-transposition of the great arteries. Staged surgical repair consisted of a Blalock–Hanlon septectomy immediately after birth and ASR according to Mustard at the age of 1 year.

She was on medical therapy consisting of low-dose aspirin and furosemide 40 mg.

She visited the outpatient clinic complaining of shortness of breath and reduced exercise capacity. Clinical examination revealed low blood pressure of 90/50 mmHg, an O_2_ saturation of 92%, a weight gain of 6 kg, elevated jugular venous pressure, bilateral lung crepitations, and peripheral oedema of the lower extremities, all suggestive of systemic and pulmonary congestion. Lab tests showed acute kidney failure with an elevated creatinine of 1.4 mg/dL (normal 0.51–0.95 mg/dL), compared to 0.6 mg/dL at baseline. N-terminal pro-B-type natriuretic peptide was also high at 10 191 ng/L (normal < 170 ng/L).

Given the clinical signs of heart failure, the patient was admitted to the intensive care unit. Ionotropic support was initiated using dobutamine, and decongestion was pursued using intravenous bumetanide. After achieving decongestion and recovery of kidney function, the four pillars of heart failure therapy were initiated: spironolactone 25 mg, bisoprolol 2.5 mg, dapagliflozin 10 mg, and sacubitril/valsartan 24/26 mg.^6^ Diuretic therapy could not be discontinued and was maintained orally using bumetanide 1 mg daily. In pursuit of an aetiology for the patient’s heart failure, a 2D transthoracic echocardiography was performed, revealing an expected dilated morphological right, systemic, ventricle with moderately decreased ejection fraction as well as an unexpected dilated morphological left, sub-pulmonary, ventricle. The echocardiography also revealed prominent turbulent flow at the level of the PVB.

**Figure 1:**
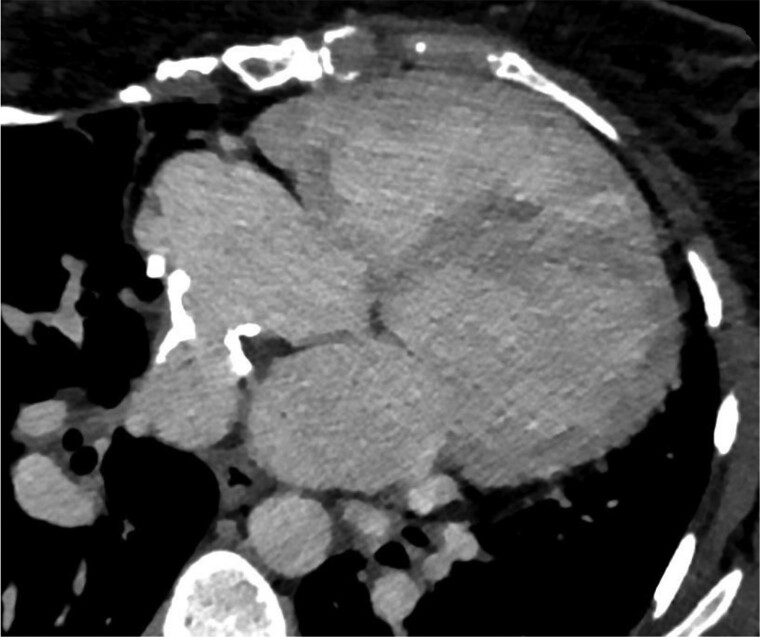
Computed tomography scan of stenotic pulmonary venous baffle.

Invasive haemodynamic evaluation revealed a pulmonary arterial pressure (AP) of 117/45/70 mmHg and a systemic ventricular pressure of 88/9 mmHg. The transpulmonary pressure gradient was 36 mmHg, calculated by subtracting the end-diastolic pressure in the systemic ventricle from the mean AP. A cardiac computed tomography showed a severely calcified and stenotic pulmonary venous baffle with a diameter of 9 mm (*Figure 1*).

The patient’s case was discussed multidisciplinary within the team of adult congenital heart disease specialists, the interventional cardiologists, and the cardiac surgeons. Conservative treatment posed the risk of low output due to decreased preload and sudden cardiac death, especially in the setting of atrial arrhythmias or during exercise.^7,8^ Furthermore, patient remained in functional class IIb after initiation of heart failure treatment. Surgical re-intervention was considered high risk because of redo sternotomy, heavily calcified intracardiac patches, and anticipated difficulties during cardiopulmonary bypass, especially in a patient with severe pulmonary hypertension and biventricular heart failure. Peri-operative mortality in this setting is reported between 21 and 36%.^3^ Additionally, there is no guarantee of long-term success following redo atrial switch surgery, with increased risk of baffle leaks and restenosis.^7^ Therefore, percutaneous intervention was preferred. In this, access to the pulmonary venous atrium must be obtained and is difficult. Access to the PVB can be obtained retrograde via femoral arterial access, but navigating large French catheters along this trajectory is cumbersome. Transseptal puncture has been described but has equally difficult access angles and leaves an iatrogenic baffle leak. Hybrid stenting through subxiphoidal incision/mini-sternotomy gives quick access to the PVB but necessitates re-sternotomy. Therefore, we chose hybrid stenting by left lateral sternotomy and through the systemic ventricular apex.

The patient was brought to the catheterization laboratory. Under general anaesthesia, an apical access was provided by the cardiac surgeon. The procedure was guided by transoesophageal echocardiography. The degree of stenosis and turbulent flow in the stenotic PVB can be appreciated in Supplementary material online, *Video S1A* and *B*.

**Figure 2:**
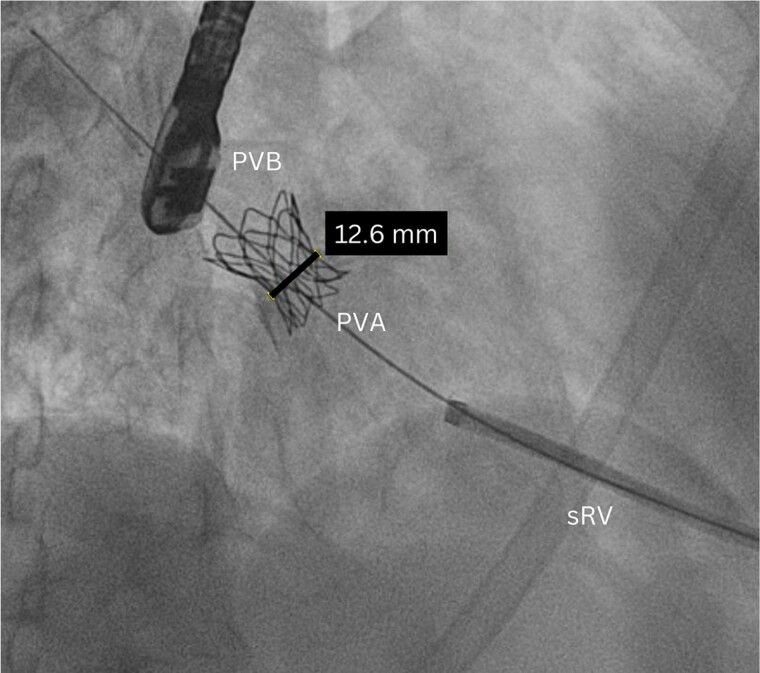
Fluoroscopy image after stenting and dilatation of the pulmonary venous baffle.

Through the apex of the systemic right ventricle, the tricuspid valve and the left neo-atrium and the pulmonary venous baffle were reached. A semi-compliant balloon was placed into the baffle, and the ostium was sized at 9 mm. A balloon-expandable covered CP stent mounted on a balloon was placed at the level of the baffle stenosis. This stent was flared proximally and distally using a semi-compliant balloon. Next, the stent was progressively dilated. There was significant recoil, but eventually, a stent waist of 13 mm was obtained (*Figure 2*). Fluoroscopy showed nice placement of the stent into the pulmonary venous baffle (see Supplementary material online, *Video S2*). Transoesophageal echocardiography showed improved and laminar flow across the stent (see Supplementary material online, *Video S3A*–*C*) with reduction of the peak instantaneous pressure gradient across the baffle stenosis from 18 to 11 mmHg. The apical access was closed by the surgeons.

The patient was weaned of the ventilator immediately after the procedure and brought to the coronary care unit for observation which remained uneventful.

The patient was on low-dose aspirin, which was continued after the procedure. No thrombotic complications were seen at 6 months of follow-up.

The patient was discharged home 2 days after the procedure.

She visited the outpatient clinic 3 and 6 months after discharge and felt remarkable improvement, with regular physical exercise and gardening. She restarted work as a secretary. On physical examination, no signs of congestion were noted. Echocardiography showed discrete turbulent flow through the stent which was measured at 14 mm with a residual peak gradient of 10 mmHg and good pulmonary venous inflow.

## Discussion

This case highlights the successful hybrid intervention by left lateral mini-thoracotomy in a challenging scenario of severe PVB stenosis in a patient with complex congenital heart disease. Surgical correction of transposition of the great arteries by ASR was developed in the early 1960s.^9,10^ When left untreated, mortality in patients born with transposition of the great arteries is 90% in the first year. Although peri-operative mortality was as high as 30% in the early experience, this dropped to 15% in patients operated after 1980. As a result, more than 70% of patients are alive at the age of 30. Clinical course and complications are well documented.^1,3,11^ However, the clinical course in these patients is a spectrum.^1,11^ Although guidelines on the management of congenital heart disease are available, management of each patient should be individualized.^12^

Over the course of a lifetime after surgery, events occur. At that time, management of the patient should be evaluated in team. In this case report, we are confronted with a patient presenting in heart failure due to stenosis of the PVB. She remained symptomatic with elevated pulmonary APs, even after initiation of heart failure treatment. It was decided that structural optimization was necessary to maintain better long-term prognosis. Surgical intervention, interventional approach, hybrid approach, and listing for heart transplantation were discussed. As the operative risk is known to be high and the surgical success was unsure, we opted for an interventional approach.

For the systemic venous baffles, percutaneous stenting and dilatation of baffle stenosis by femoral venous access are the first choice.^5,13,14^ However, in this case involving a PVB, the challenge lies in accessing the obstruction. One could consider attempting a retrograde approach from the femoral artery working our way up through the aortic and tricuspid valve or gaining access through a transseptal puncture across the baffle. However, both methods present challenges for achieving optimal stenting in this context: the former due to its time-consuming nature as well as the anticipated difficulty passing a larger French size sheath along this trajectory and the latter due to the complexity of navigating sharp angles as well as a residual iatrogenic baffle leak.^14^ A hybrid approach utilizing surgical access for stent placement and dilatation has been published as well.^4^ Here, the authors achieved catheter access to the pulmonary venous atrium via a subxiphoid approach. However, this method required a partial sternotomy. In this patient, we chose a hybrid transcatheter approach using a left lateral mini-thoracotomy and apical systemic ventricular access. The PVB could be accessed for stenting and dilation through the tricuspid valve. This way, subxiphoid incision and partial sternotomy are avoided and a straight path with the large French sheath across the stenosis is established.

As patients undergoing stenting of the PVB are scarce, post-interventional treatment is often individualized. Generally, after baffle stenting, aspirin monotherapy is sufficient with no cases of stent thrombosis reported, but dual-antiplatelet therapy with aspirin/clopidogrel or resumption of anticoagulation in combination with aspirin are reported as well.^5,15^ From the treatment of pulmonary venous stenosis, we have learned that stent diameter is an important contributor to stent patency.^16^ In this, dual-antiplatelet treatment with aspirin/clopidogrel during the first 3–6 months with aspirin monotherapy is generally favoured.^16^ Because of the obtained diameter of the stent and given that flow through the stent consists of the entire cardiac output, we consider aspirin monotherapy as sufficient. During follow-up, no stent thrombosis has been observed.

In conclusion, this case shows the feasibility of access to the pulmonary venous atrium by left lateral mini-thoracotomy and through systemic ventricular apical access. Furthermore, it highlights the importance of pre-interventional planning and the multidisciplinary collaboration between adult congenital cardiologist, the echocardiographer, and the innovative techniques employed, underscoring the importance of individualized approaches in the management of such cases.

## Lead author biography


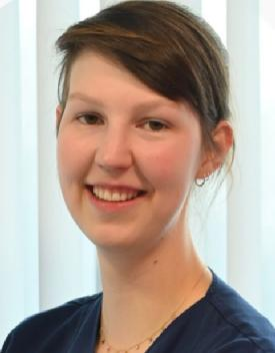


Dr Margaretha Van Kerrebroeck earned her medical degree with great distinction from the Catholic University of Leuven in 2018. She is currently a trainee in cardiology at the University Hospitals of Leuven with a specific focus on heart failure, echocardiography, and intensive care.

## Supplementary Material

ytae616_Supplementary_Data

## Data Availability

The data underlying this article are available in the article and in its online Supplementary material.

## Notes

**Consent:** The authors confirm that consent for submission and publication of this case report including images and associated text has been received from the patient in line with the Committee on Publication Ethics (COPE) guidelines.

**Funding:** None declared.
